# Ribitol and ribose treatments differentially affect metabolism of muscle tissue in FKRP mutant mice

**DOI:** 10.1038/s41598-024-83661-4

**Published:** 2025-01-08

**Authors:** Marcela P. Cataldi, Qi L. Lu

**Affiliations:** 1 McColl-Lockwood Laboratory for Muscular Dystrophy Research, Carolinas Medical Center, Atrium Health Musculoskeletal Institute, 1000 Blythe Blvd. , Charlotte, NC 28231 USA; 2 McColl-Lockwood Laboratory for Muscular Dystrophy Research, Carolinas Medical Center, Atrium Health Musculoskeletal Institute, 1000 Blythe Blvd., Charlotte, NC 28203 USA

**Keywords:** Neuromuscular disease, Metabolomics

## Abstract

Dystroglycanopathy is characterized by reduced or lack of matriglycan, a cellular receptor for laminin as well as other extracellular matrix proteins. Recent studies have delineated the glycan chain structure of the matriglycan and the pathway with key components identified. FKRP functions as ribitol-5-phosphate transferase with CDP-ribitol as the substrate for the extension of the glycan chain. Supplement of ribitol and ribose have been reported to increase the levels of CDP-ribitol in both cells and in muscles in vivo. Clinical trials with both ribitol and ribose have been reported for treating LGMD2I caused by mutations in the FKRP gene. Here we compared the comprehensive metabolite profiles of the skeletal muscle between ribitol-treated and ribose-treated FKRP mutant mice. The closely related pentose and pentitol show clearly differential impacts on metabolisms despite their similarity in enhancing the levels of CDP-ribitol and matriglycan synthesis. Supplement of ribitol changes lysophospholipid sub-pathway metabolite profiling with a trend towards normalization as reported in the muscle after AAV9-FKRP gene therapy. Ribose treatment significantly increases level of ribonate and elevates levels of advanced glycation end products. Further analysis is required to determine which metabolite is prudent to use for long-term daily treatment of dystroglycanopathies.

## Introduction

*O*-mannosylation of alpha dystroglycan (α-DG) is conserved in vertebrates^1,2^. This O-mannosylation produces the laminin-binding glycan, matriglycan, which acts as a cellular receptor for laminin as well as other extracellular matrix (ECM) proteins, including agrin, perlecan, neurexin, and pikachurin^3–8^. This linkage from ECM proteins through matriglycan to the membrane-bound dystrophin protein complex, and further to cytoskeleton proteins, is critical for neuronal development and muscle integrity and functions^9,10^. Dystroglycanopathy is characterized by reduced or lack of matriglycan. Hypoglycosylation of α-DG reduces its binding strength to ligand proteins in the ECM, weakening integrity of muscle structure and leading to damage of muscle fibers under contraction-related stress. This leads to progressive loss of muscle fibers and functions in both cardiac and skeletal muscles.

To date, mutations in at least eighteen genes have been associated with hypoglycosylation of α-DG. Among them, mutations in the gene encoding for the fukutin-related protein (FKRP) are the most common causes of dystroglycanopathy^11,12^. Recently, the structure of the laminin-binding matriglycan has been delineated with the following chain: (3GlcA-β1-3Xyl-α1) *n*-3GlcA- β1-4Xyl-Rbo5P-1Rbo5P-3GalNAc- β1-3GlcNAc- β1-4(P-6) Man-1-Thr/ser^13–15^. The glycan chain extension pathway is completed by the following proposed transferase activity: POMT1 and POMT2 catalyze the initial *O-*mannosylation of the proteins^16^. Further extension of the sugar chain is carried out by POMGnT2 (GTDC2)^17,18^, B3GALNT2^19^, FKTN, FKRP^13^, TMEM5^20^ and B4GAT1 successively^21^. Finally, LARGE acts as a bifunctional glycosyltransferase having both xylosyltransferase and glucuronyltransferase activities, producing repeated units of 3GlcA-1-3Xyl-1^22^. Furthermore, matriglycan synthesis pathway studies also identified isoprenoid synthase domain containing (ISPD) as a cytidyltransferase (pyrophosphorylase) producing CDP-ribitol^13–15,23^. CDP-ribitol is the substrate of FKRP and Fukutin (FKTN) for the extension of the glycan chain of α-DG with ribitol-5-phosphate (ribitol-5P)^15,23^. Interestingly, Gerin et al.. demonstrated that ribitol and ribose treatment of HEK293 cells overexpressing ISPD and patient-derived ISPD-deficient fibroblasts leads to an increase of CDP-ribitol levels and partially corrects the defect in glycosylation of α-DG caused by ISPD mutation^15^. These results suggest that the levels of CDP-ribitol might be one of the limiting factors of this *O*-mannosylation and the supplement of ribitol or ribose can increase the levels of CDP-ribitol, thus enhancing synthesis of matriglycan as a treatment for dystroglycanopathies caused by mutations of FKRP. We hypothesize this is possible as most mutant FKRPs retain at least partial function^24^. An increase in the levels of CDP-ribitol substrate might enhance the efficiency of remaining function of mutant FKRP, thus compensating for the reduced function of mutant FKRPs and enhancing matriglycan synthesis. Indeed, in vivo study in dystroglycanopathy mouse model with FKRP mutations confirms that ribitol treatment is able to increase the levels of CDP-ribitol and partially restore the hypoglycosylation of α-DG in both skeletal and cardiac muscles with improvement in muscle functions^25^. Importantly, ribitol treatment showed no effect on liver, kidney and spleen histology, as well as liver and kidney function. This has led to the ongoing clinical trials of ribitol for LGMD2I (NCT04800874 and NCT05775848). The potential of using ribose for treatment of dystroglycanopathies has also been considered due to its nature as metabolite and as over-the-counter availability to use as supplement with low cost. More recently, a study reported a 6-month oral supplement of ribose in a single case of LGMD2I patient with homozygous common L276I mutation^26^. Ribose is well tolerated in doses up to 18 g/day. The treatment resulted in a decrease of serum creatine kinase levels. Although objective improvement in clinical and patient-reported outcome measurement is not observed, the patient reported subjective improvement of muscle strength, fatigue, and pain.

Both ribitol and ribose are normal metabolites in vertebrates and likely to have effect on matriglycan expression with similar mechanisms. However, ribose and ribitol have distinct roles in cellular metabolism and produce different metabolic footprints. Ribose is crucial for RNA and energy production whereas little is known about ribitol except that it is part of the chemical structure of riboflavin (vitamin B) and flavin mononucleotide (FMN), which are nucleotide coenzymes used by many enzymes (flavoproteins) in various metabolic pathways^27^. Perhaps also important is that ribose is known to induce advanced glycation end products (AGEs) and protein aggregation both inside and outside cells^28,29^. Ribitol is a reduced form of pentose sugar and expected to have greatly diminished capacity for glycation of cellular molecules. Importantly, the chronic and progressive nature of dystroglycanopathies requires a life-long treatment with these metabolites. Our earlier analyses by metabolomics in cells in vitro also show differential effect of ribitol and ribose well beyond the matriglycan restoration^30^. In this study, we compare the effects of ribitol and ribose on muscle cell metabolism using the FKRP^P448L^ mutant mice (P448L) as an in vivo model system by applying untargeted global metabolomics profiling. This analysis will help us to better understand the metabolic impact of the pentose and pentitol as drugs for muscular dystrophy and provide potential biomarkers for evaluating efficacy and safety of the treatments. The results show that the two chemically closely related pentose and pentitol have differential impacts on metabolisms despite their similarity in enhancing the levels of CDP-ribitol and matriglycan synthesis in the muscles. Supplement of ribitol changes lysophospholipid sub-pathway metabolite profiling with a trend towards normalization as reported in the muscle with AAV9-FKRP gene therapy. A surge in level of ribonate and elevated levels of AGEs with ribose treatment require further evaluation as a potential long-term daily treatment for dystroglycanopathies.

## Results

### Treatment regime and summary of global metabolomic profiling

We treated P448L mutant breeding females with 10% ribitol or 10% ribose in drinking water when they became pregnant and continued the treatment to the pups until they reached 32 weeks of age. The mice were terminated, and the quadriceps muscles were collected for metabolomics analyses. Both ribitol and ribose were well tolerated with no death due to the treatment. This is supported by the body weight measurement at 30 weeks of age, showing no significant difference between ribitol or ribose-treated mice compared to the control mice, although a slight increase on mean weight was detected in the treated male (Fig. S1). Four muscle samples (2 females and 2 males) for each cohort (untreated control, ribitol-treated and ribose-treated) were examined for untargeted global metabolic profiling (UGMP) by Ultrahigh Performance Liquid Chromatography-Tandem Mass Spectroscopy (UPLC-MS/MS).

**Fig. 1:**
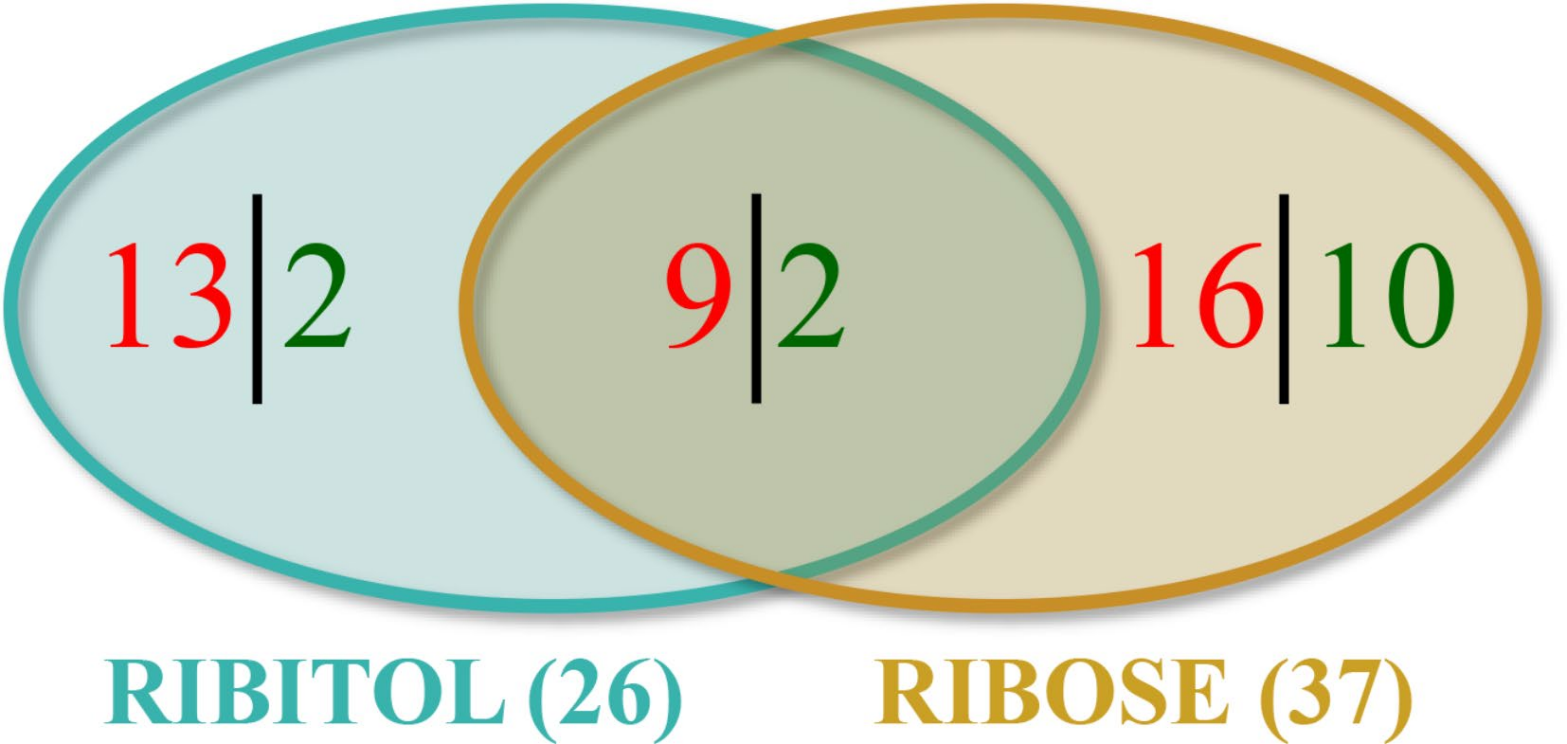
Venn diagram illustration of number of metabolites with significant changes in levels shared and differentially affected by ribitol and ribose treatments when compared to the control. Samples were from 32-week-old P448L mice treated with 10% ribitol or 10% ribose and age-matched untreated P448L control mice. Welch’s two-sample t-test, p *≤* 0.05. Red and green indicate the number of upregulated and downregulated metabolites, respectively.

The analysis identified and quantitated a total of 568 metabolites with known identity. Ribitol or ribose treatment showed statistically significant changes (*p* ≤ 0.05) in almost all major metabolic pathways, accounting for 4.6% and 6.5% of total detected metabolites when compared to the untreated P448L mice respectively (Table 1). These included amino acid (6 and 10 metabolites), carbohydrate (4 and 7), energy (1 and 0), lipid (7 and 8), nucleotide (4 and 3), cofactor/vitamin (1 and 8), and xenobiotic (3 and 1) with ribitol and ribose treatment, respectively (Table S1). Furthermore, most of the affected metabolites were upregulated (22 and 25 with ribitol and ribose treatment, respectively) compared to the untreated controls, bearing a total of 84.6% and 67.6% of metabolites with significant changes by ribitol and ribose treatment, respectively. Out of the 22 and 25 upregulated metabolites, only 9 were increased by both treatments (Fig. 1). There were 4 and 12 metabolites downregulated by ribitol and ribose treatment, respectively, but only 2 were decreased by both treatments. This indicates a differential effect of the two sugars on metabolism. Statistically significant differences were also detected on 13 metabolites (2.3% of total detected metabolites) when the muscles of ribose-treated mice were compared to ribitol-treated mice (Table 1).

**Table 1:** Summary of the global metabolic profiling.

Metabolic pathway	Metabolites assayed	Ribitol vs control	Ribose vs control	Ribose vs ribitol
Amino acid	132	6	10	2
Peptide	17	0	0	0
Carbohydrate	44	4	7	1
Energy	12	1	0	1
Lipid	285	7	8	5
Nucleotide	34	4	3	1
Cofactor/vitamin	25	1	8	1
Xenobiotic	19	3	1	2
Total	568	26	37	13
% of total		4.6%	6.5%	2.3%
Upregulated/downregulated		22|4	25|12	8|5

Numbers represent total metabolites achieving statistical significance (Welch’s two-sample t-test, p<0.05) detected in each sub-pathway and compared between the groups shown. Samples analyzed for untargeted global metabolic profiling were quadriceps muscles derived from 32 weeks of untreated control and 10% ribitol or 10% ribose treated P448L mice.

### Effect of ribitol and ribose on carbohydrate metabolites: both treatments increase the levels of ribitol, ribitol-5-P and CDP-ribitol, but differentially affect the levels of ribonate, arabonate and xylonate

**Fig. 2:**
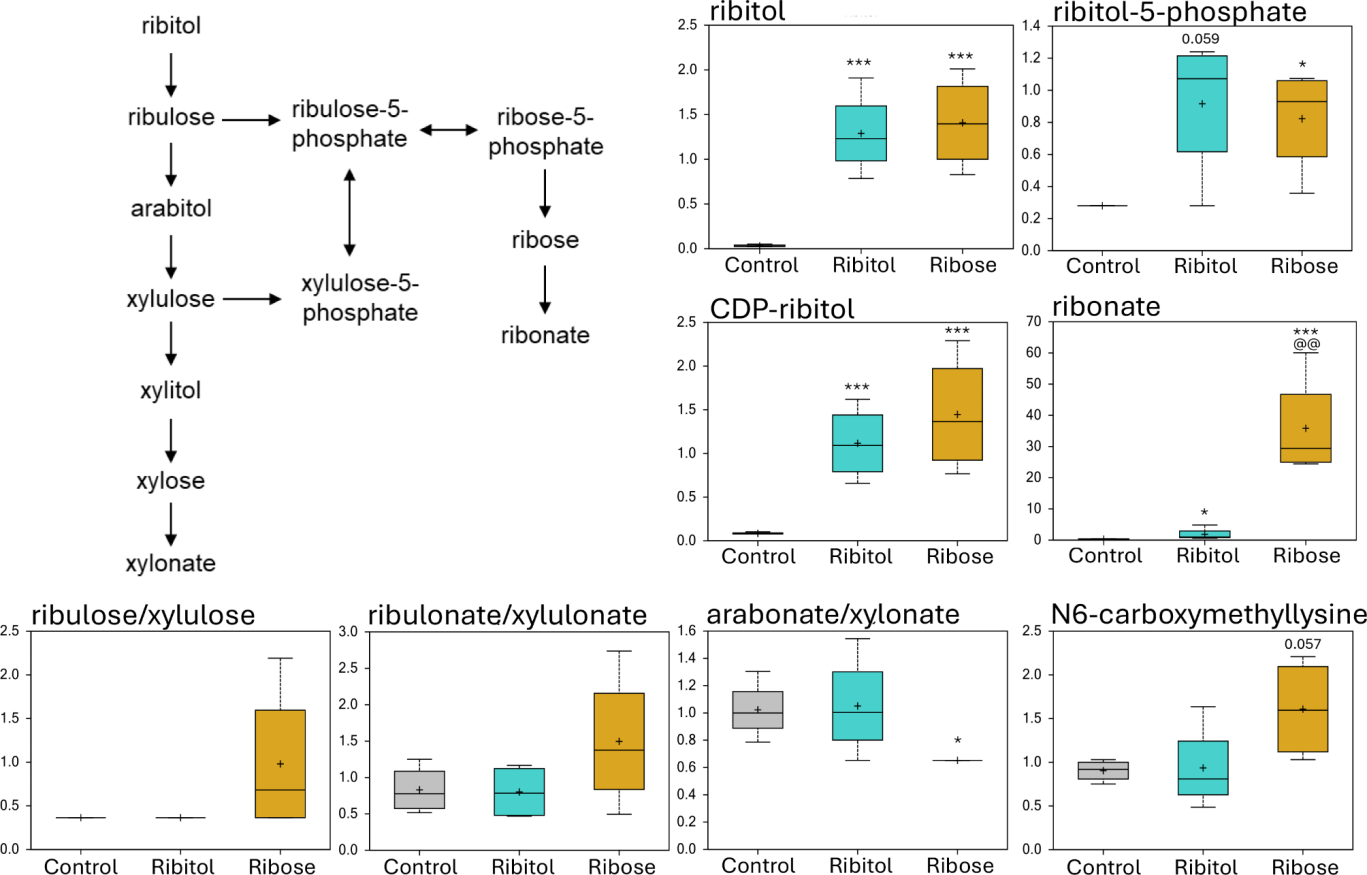
Carbohydrate metabolism. Pentose/pentose phosphate pathway. Comparison of metabolite abundances in quadriceps from 32-week-old control P448L mice, and 10% ribitol or 10% ribose treated P448L mice. Vertical axis represents scaled intensity in arbitrary units. *p ≤ 0.05, ** *p* ≤ 0.01, *** *p* ≤ 0.001 compared to the control. @@*p* ≤ 0.01 compared to ribitol treatment, as determined by Welch’s two-sample t-test.

As expected from earlier results of in vitro and in vivo studies, both ribitol and ribose treatments led to significantly higher levels of cellular ribitol (40.42- and 44.13-fold increase with ribitol and ribose treatment, respectively), ribitol 5-P (3.27 and 2.94-fold increase with ribitol and ribose treatment, respectively) and CDP-ribitol (13.19 and 17.11-fold increase with ribitol and ribose treatment, respectively) when compared to the control (Fig. 2 and Table S1). The difference in levels of the 3 metabolites between the two treatments is not significant. Interestingly, one of the most significant changes is the level of ribonate, which is upregulated by both treatments when compared to the control. However, the fold increase is 117.79 for the ribose-treated muscles while only 6.09 for the ribitol-treated muscles over the controls. The difference between the two treated groups is also statistically significant. The high amount of ribonate is likely the oxidized derivative of the ribose, which is not detected by the study measurement. Differential effect among other compounds from the pentose metabolism pathway includes the increase in ribulose/xylulose and ribulonate/xylulonate in the ribose-treated samples but not in the ribitol-treated samples compared to the control (Fig. 2). Furthermore, arabonate/xylonate levels showed statistically significant reduction in the ribose-treated samples, but not in the ribitol-treated samples compared to the control. Ribitol and ribose treatment also show differential effect on levels of other metabolites in the glycolysis pathway. The levels of 3-phosphoglycerate and phosphoenolpyruvate (PEP) were clearly reduced with ribitol treatment, but not with ribose treatment. However, no change was detected for glucose, glucose-6-phosphate, fructose-6-phosphate, lactate and pyruvate with both treatments (Fig. S2). Importantly, the levels of N6-carboxymethyllysine, an AGE product, was clearly higher in the ribose treated group, 1.72- and 1.78-fold over the ribitol-treated and the control group, respectively (Fig. 2 and Table S1). The potential significance of the change for clinical application will be reviewed in the Discussion.

### Effect of ribitol and ribose treatment on tricarboxylic acid cycle (TCA)

**Fig. 3:**
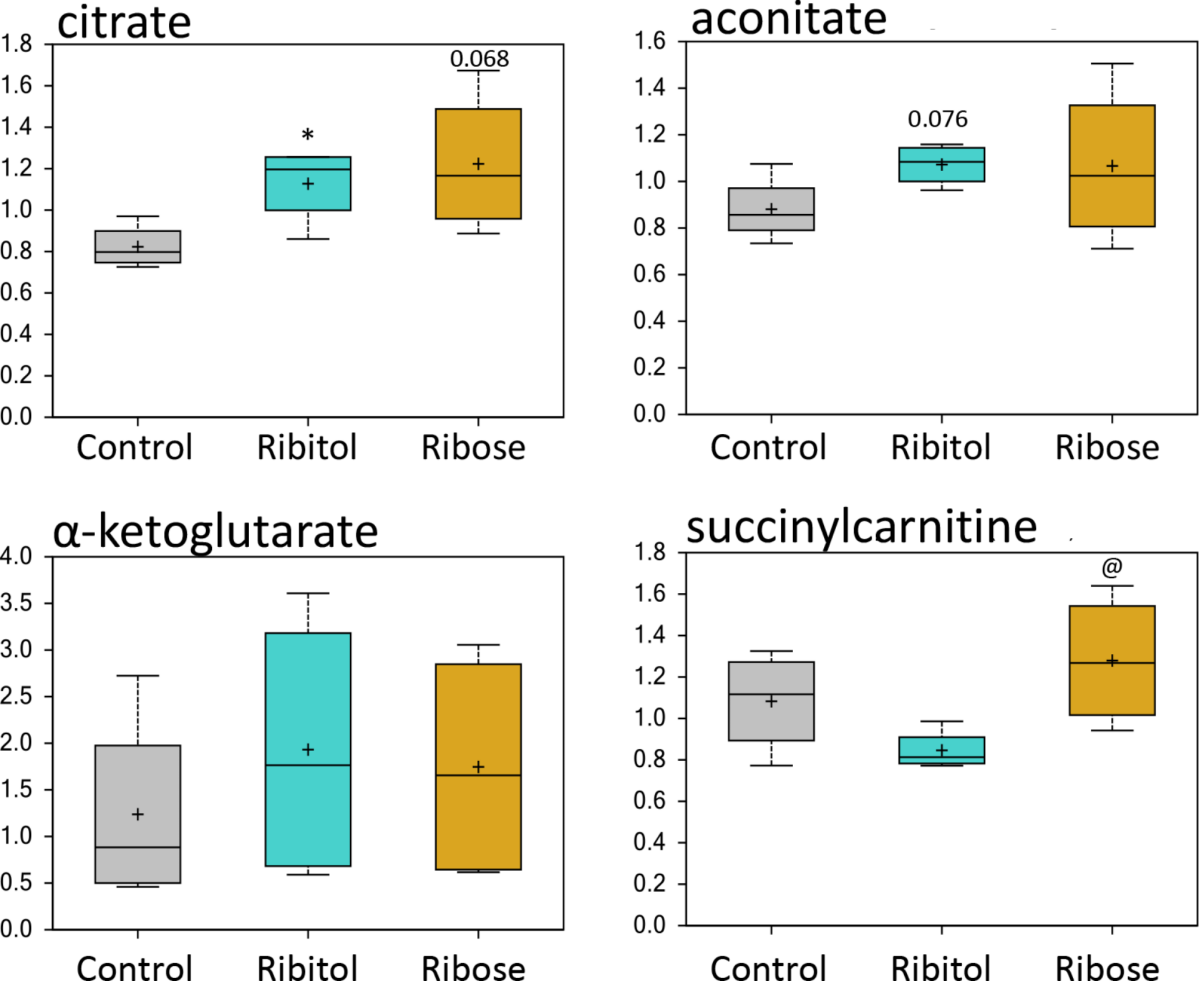
TCA cycle. Comparison of metabolite abundances in quadriceps from 32-week-old control P448L mice, and 10% ribitol or 10% ribose treated P448L mice. Vertical axis represents scaled intensity in arbitrary units. * *p* ≤ 0.05 compared to the control; @ *p* ≤ 0.05 compared to ribitol treatment as determined by Welch’s two-sample t-test.

The level of citrate was clearly upregulated by both treatments, although the increase by ribose treatment barely missed statistical significance (Fig. 3). Levels of aconitate (cis or trans) and α-ketoglutarate were also slightly increased with both treatments. Levels of succinylcarnitine (C4-DC) was slightly reduced with ribitol treatment but increased with ribose treatment when compared to the control, leading to statistical significance between the two treatment groups (Fig. 3 and Table S1). No significant change was detected for other TCA related metabolites including malate, isocitrate, fumarate and succinate (Fig. S3).

### Effect of ribitol and ribose treatment on amino acid and protein metabolisms

**Fig. 4:**
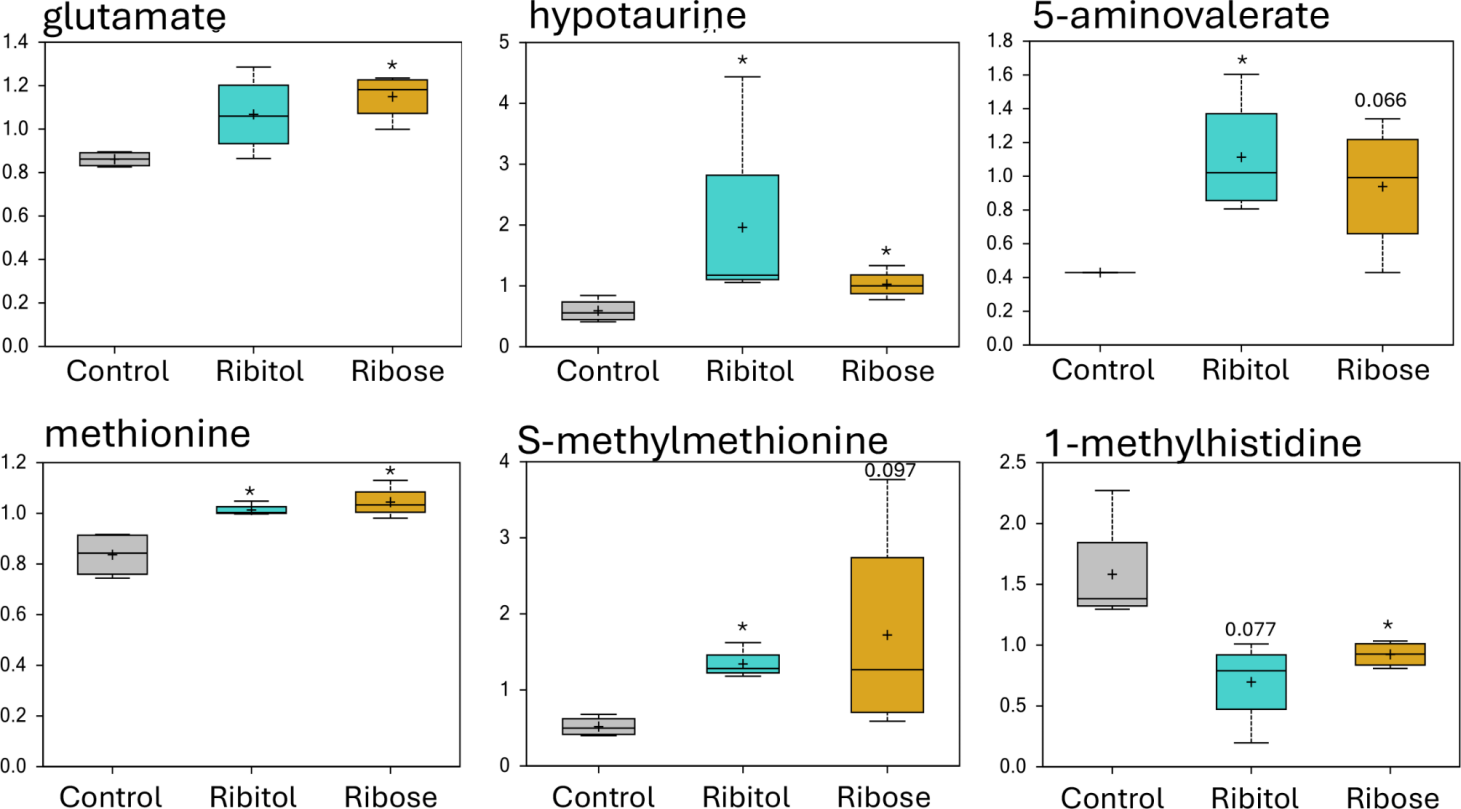
Amino acid metabolism. Comparison of metabolite abundances in quadriceps from 32-week-old control P448L mice, and 10% ribitol or 10% ribose treated P448L mice. Vertical axis represents scaled intensity in arbitrary units. *p ≤ 0.05 compared to the control as determined by Welch’s two-sample t-test.

Among the detected 132 metabolites involved in the amino acid metabolic pathways, 6 and 10 metabolites reached statistical significance (*p* ≤ 0.05) after ribitol and ribose treatment respectively when compared to the control group (Table S1). There were 2 metabolites, methionine sulfoxide and 1-methylguanidine, showed significant difference between the two treatment groups (Table S1). Specifically, the majority metabolites related to the sub-pathways of glycine, serine and threonine metabolism exhibited higher measurement after ribitol and ribose treatment. However, only sarcosine and dimethylglycine reached statistical significance (*p* ≤ 0.05) after ribose treatment, and threonine after both treatments when compared to the control group (Fig. S4). Metabolites in most other sub-pathways of amino acid, such as alanine, aspartate, glutamate, histidine, lysine, phenylalanine, tyrosine, tryptophan, leucine, isoleucine, valine, methionine and cysteine metabolism show limited changes between the 2 treated groups and control. However, 5 metabolites (glutamate, hypotaurine, 5-aminovalerate, methionine and S-methylmethionine) were significantly increased (or approached significance) by both treatments, while 1-methylhistidine was downregulated with both treatments (Fig. 4). Moreover, from the urea cycle sub-pathway, urea and citrulline were increased by both treatments when compared to the control (Fig S5 and Table S1). The levels of the majority of the 17 metabolites identified in the peptide metabolism, including the gamma-glutamyl amino acid, dipeptide, and acetylated peptide sub-pathways were higher with both ribitol and ribose treatments compared to the controls, but without significance between the groups (Table S2). The significance of these changes remains to be explained.

### Effect of ribose and ribitol on lipids metabolism

**Fig. 5:**
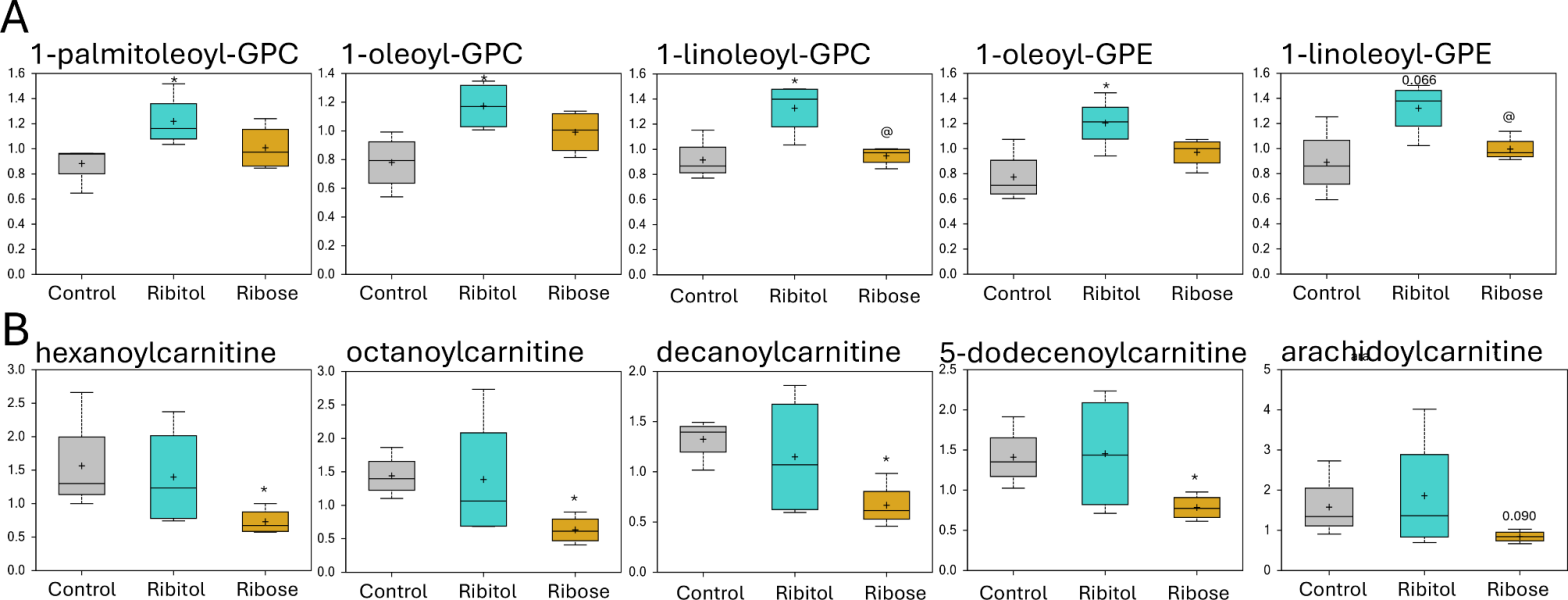
Metabolism of lipids. Comparison of metabolite abundance in quadriceps from 32-week-old control P448L mice and 10% ribitol or 10% ribose treated P448L mice. Vertical axis represents scaled intensity in arbitrary units. (**A**) Metabolites involved in lysophospholipid sub-pathway. (**B**) Metabolites involved in fatty acid metabolism (acylcarnitine). *p ≤ 0.05 compared to the control; @*p* ≤ 0.05 compared to ribitol treatment as determined by Welch’s two-sample t-Test.

Half (285 metabolites) of the total metabolites detected in this study are involved in the lipid metabolic super-pathway. However, only 7 and 8 of them showed statistical significance when ribitol and ribose treated mice were compared to control, respectively (Table S1). Interestingly, while ribitol treatment mostly upregulated the affected metabolites compared to control (6 out of 7), ribose mostly lowered the levels of the metabolites (7 out of 8). Only 1 out of 20 detected phosphatidylcholines (PC) shows significantly higher levels in the muscles treated with ribitol, but none with ribose when compared to control (Table S1). However, ribitol treatment clearly increased the levels of nearly all (24 out of 27) lysophospholipid metabolites with 1 approaching (p *≤* 0.1) and 4 reaching statistical significance (p *≤* 0.05) compared to the control. These 4 metabolites are 1-palmitoleoyl-GPC, 1-oleoyl-GPC, 1-linoleoyl-GPC, and 1-oleoyl-GPE (Table S1 and Fig. 5A) (significance reviewed on discussion). In contrast, significant difference was not obtained for any of the lysophospholipids between ribose-treated and the control group, and the levels of 13 metabolites were below the levels of the control group. Most of the metabolites with significant change by ribose treatment belonged to the family of acylcarnitines, which are functionally involved in β-oxidation of fatty acid. Specifically, acylcarnitines with medium chain-length (6 to 12 carbons) including hexanoylcarnitine, octanoylcarnitine and decanoylcarnitine were downregulated in the ribose treatment groups but not in the ribitol treatment group when compared to the control group (Fig. 5B).

### Effect of ribose and ribitol on metabolism of nucleotide synthesis

**Fig. 6:**
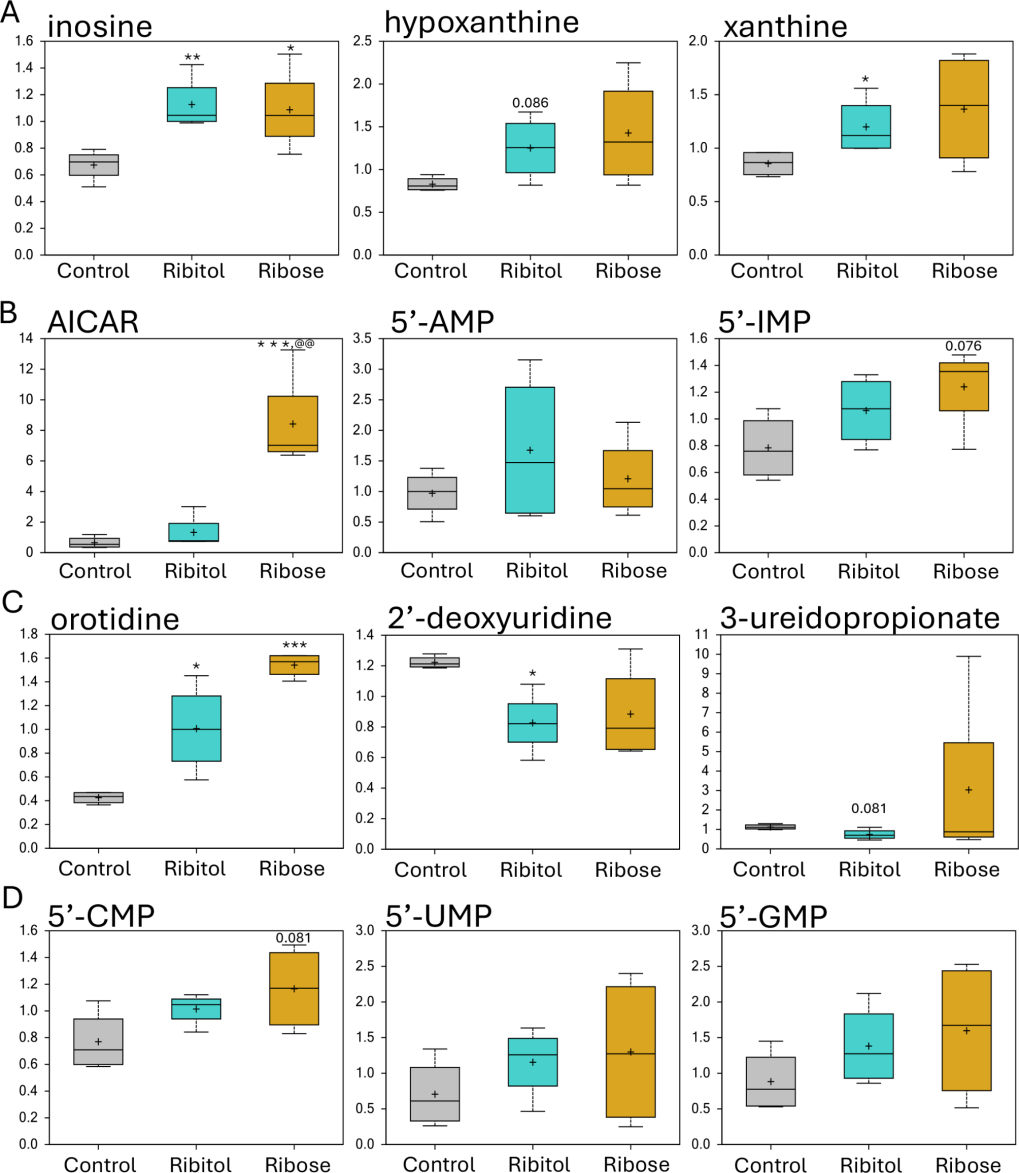
Nucleotide metabolism. Comparison of metabolite abundance in quadriceps from 32-week-old control P448L mice and 10% ribitol or 10% ribose treated P448L mice. Vertical axis represents scaled intensity in arbitrary units. (**A**) Purine metabolism sub-pathway [(Hypo) Xantine/Inosine containing]. (**B**) 5-Aminoimidazole-4-carboxamide ribonucleotide (AICAR), adenosine monophosphate (AMP), inosine 5’-monophosphate (IMP). (**C**) Pyrimidine metabolism sub-pathway (orotate and uracil containing). (**D**) Cytidine 5’-monophosphate (5’-CMP), uridine 5’-monophosphate (5’-UMP), guanosine 5’-momophosphate (5’-GMP). *p ≤ 0.05, ** *p* ≤ 0.01, ***p ≤ 0.001 compared to the control. @@ *p* ≤ 0.01 compared to ribitol treatment as determined by Welch’s two-sample t-test.

Purine and pyrimidine metabolites were in general increased by ribitol and ribose treatment when compared to the controls. From the purine metabolism sub-pathway, inosine, hypoxanthine and xanthine were elevated by both treatments compared to the control, with inosine reaching statistical significance (Fig. 6A). Interestingly, 5-Aminoimidazole-4-carboxamide ribonucleotide (AICAR) showed a 12.9-fold increase in the ribose treated samples, but only 2.0-fold increase in the ribitol treated samples compared to control, leading to a significant difference between the two treatment groups. AICAR is an intermediate for generation of inosine 5’-monophosphate (5”-IMP) and an analog of adenosine 5’-monophosphate (5’-AMP) that is important for synthesis of ATP. However, levels of 5’-AMP were not significantly changed over the control (Fig. 6B). The Level of 5’-IMP was increased by both treatments, approaching significance in ribose-treated samples (0.05 < *p* < 0.1). Of the pyrimidine metabolism sub-pathway, only one metabolite, orotidine, reached statistical significance by both treatments, with 2.3- and 3.6-fold increase above control level for ribitol and ribose treatment, respectively (Fig. 6C). Interestingly, two uracil containing pyrimidine metabolites, 2’-deoxyuridine and 3-ureidopropionate were downregulated by ribitol, with *p* < 0,05 and *p* < 0,1, respectively. Cytidine 5’-monophosphate (5’-CMP) was also elevated with both treatments, approaching significance with ribose treatment compared to the control (0.05 < *p* < 0.1) (Fig. 6D). Overall, levels of all 5’-CMP, 5’-IMP, 5’-AMP, guanosine 5’-momophosphate (5’-GMP) and uridine 5’-monophosphate (5’-UMP) were elevated with both treatment (Fig. 6B and D).

### Effect of ribose and ribitol on metabolism of cofactors, vitamins and xenobiotics

**Fig. 7:**
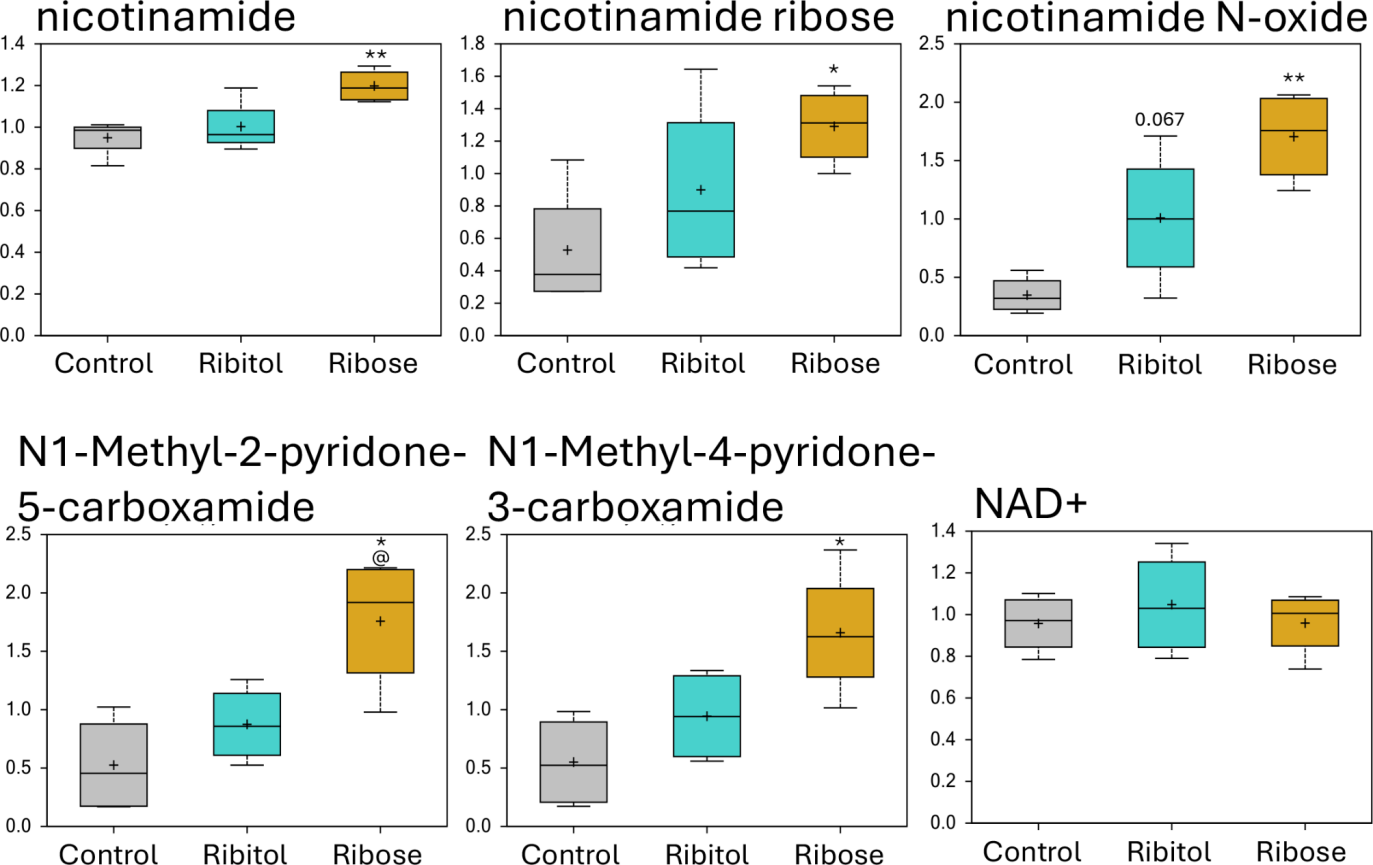
Cofactor and vitamin metabolism. Comparison of metabolite abundance in quadriceps from 32-week-old control P448L mice and 10% ribitol or 10% ribose treated P448L mice. Vertical axis represents scaled intensity in arbitrary units. *p ≤ 0.05, ** *p* ≤ 0.01 compared to the control and @ p ≤ 0.05 compared to ribitol treatment as determined by Welch’s two-sample t-Test.

Out of the 25 detected metabolites involved in cofactors and vitamins metabolism, 8 showed significance with ribose treatment compared to control. Interestingly, most of these metabolites were involved in the nicotinate and nicotinamide metabolism, including nicotinamide, nicotinamide riboside, nicotinamide N-oxide, N1-Methyl-2-pyridone-5-carboxamide and N1-Methyl-4-pyridone-3-carboxamide, and all of them were upregulated by ribose (Fig. 7). These metabolites were slightly increased by ribitol treatment without reaching statistical significance. However, the level of Nicotinamide adenine dinucleotide (NAD+) was not changed with either treatment. Similarly, thiamin (vitamin B1) from the thiamine metabolism sub-pathway, and pyridoxamine phosphate from the vitamin B6 sub-pathway were also significantly increased only with ribose treatment (Table S1). Thiamin diphosphate (TDP) was the only downregulated metabolite of all the significantly modified metabolites from the cofactors and vitamins super-pathway, and it was equally modified by both treatments compared to the control mice (Table S1). TDP is involved in the thiamine metabolism and function as cofactor for enzymes involved in carbohydrate metabolism including transketolase, α-ketoglutarate dehydrogenase and pyruvate dehydrogenase. In contrast, metabolites within the xenobiotics super pathway, benzoate and 3-phenylpropionate (hydrocinnamate) were significantly increased only with the ribitol treatment, leading to significant difference between the two treatment groups (Fig. S6). In contrast, also from the xenobiotic super pathway, stachydrine was the only metabolite which was significantly enhanced with both treatments. The effects of the two sugars on these sub-pathways are clearly differentiated, but the significance remains to be investigated.

## Discussion

We earlier reported that AAV9-FKRP treatment systemically at 5 × 10^13^ vg/kg body weight restores almost normal levels of matriglycan with significant improvement in pathology and muscle function in the P448L mutant mice^31^. The quadriceps of the AAV9-FKRP-treated mice were also analyzed by global metabolomic profiling in comparison with the control and C57BL/6 wild type mice^32^. The phosphorylcholine (GPC) and phosphorylethanolamine (GPE) were lower in the untreated control P448L mutant mice compared to the wild type mice (13 out of 15 metabolites, with 10 reaching statistical significance). AAV9-FKRP gene therapy significantly elevated the levels of most of these metabolites, with 11 out of 15 metabolites showing statistically significant difference when compared to the untreated control. The restored levels of the metabolites reached those measured in the wild type C57 mice. We suggested that increasing levels of lysophospholipid metabolites as GPC and GPE could be useful biomarkers of therapeutic effect. In the current study, the same muscles were analyzed by the same company using the same UPLC-MS/MS technique as used in the AAV9-FKRP study. Nearly the same number of metabolites with similar number for each pathway and sub-pathways were detected and measure between the two studies. Interestingly, ribitol treatment also elevates 14 out of 15 lysophospholipid metabolites associated with GPC and GPE. Perhaps more importantly, 4 significantly (p *≤* 0.05) elevated metabolites, 1-oleoyl–GPC, 1-palmitoleoyl-GPC, 1-linoleoyl-GPC, 1-oleoyl-GPE are shared with both AAV9-FKRP gene therapy and ribitol treatment. However, none of these metabolites reaches statistical significance between ribose treatment group and the control mice. This result may suggest that ribitol treatment is more potent in restoration of normal metabolisms. Also interesting is that two metabolites of glycolysis pathway, 3-phosphoglycerate and PEP were elevated in the control P448L mice compared to C57 mice, but significantly reduced in the muscles with both AAV9-FKRP therapy (*p* < 0.05) and ribitol treatment (p *≤* 0.1), but not with ribose treatment compared to the untreated P448L, further indicating a differential effect of the two metabolites.

One concern related to long-term daily taking of relatively high doses of sugars is the glycation of proteins, nucleic acid or lipids. This occurs when reducing sugars react with cellular and blood biomolecules in the environment, producing the advanced glycation end products (AGEs). AGEs can cause damage to the cellular structure altering its function and have been implicated in aging and the development or worsening of many degenerative diseases, such as diabetes, atherosclerosis, chronic kidney disease, and Alzheimer’s disease^33^. Therefore, AGEs can be considered as biomarkers for diseases prevention and intervention. Ribose is a critical component of cellular metabolism and involved in many important metabolic pathways. However, ribose is also a potent reducing agent producing AGEs and impairing the function of the target molecules^34,35^. The glycation potential of ribose to the skeletal muscle is expected and now demonstrated with the detection of greatly increased levels of AGEs as N6-carboxymethyllysine in the ribose treated muscle, but not in the ribitol treated muscles when compared to the control P448L mice. These results also suggest that monitoring alteration in metabolites and glycation status should be considered for the metabolite based long term intervention in clinical trials. Furthermore, ribose treatment results in levels of ribonate 117- and 19-fold higher than those in the control and ribitol treatment group, respectively. This extremely high levels of ribonate in the ribose treated muscle could be a concern as it has been associated with mortality in chronic kidney disease^36^.

These results together emphasize that understanding the patterns and differential effect of these metabolites may be critical for rational design of their supplementation for different conditions.

## Methods

### Animal care

All methods were performed in accordance with the Office of Laboratory Animal Welfare guidelines for the humane care and use of experimental animals, and all studies were approved by the Institutional Animal Care and Use Committee (IACUC) of Carolinas Medical Center (Charlotte, NC) and Wake Forest University. Animals were housed in the viviarium of Carolinas Medical Center. Animals were ear tagged before their group assignment. Food and water were available ad libitum during all phase of the study. Body weight was measured from 4 weeks of age until experimental endpoint. This study is reported in accordance with ARRIVE guidelines (https://arriveguidelines.org).

### Mouse models and experimental procedure

FKRP P448L mutant mice were generated by the McColl-Lockwood Laboratory for Muscular Dystrophy Research, as previously described^37,38^. These mice contain a homozygous missense mutation (c.1343 C > T, p.Pro448Leu) in the *FKRP* gene with the floxed neomycin resistant (Neo^r^) cassette removed from the insertion site. FKRP P448L mice become symptomatic at a very young age (approximately 3–4 weeks) and display a mild-to-moderate phenotype throughout the lifespan.

Ribitol and D-Ribose were purchased from Biosynth International (Itasca, IL) and dissolved in drinking water to a final concentration of 10%. Water bottles containing ribitol or ribose were changed once per week. P448L breeding females drank 10% ribitol or 10% ribose in drinking water when they became pregnant, and their pups continued drinking water supplemented with the same sugar until they reached 32 weeks of age. Mice were anaesthetized with inhalation of 1–5% Isoflurane mixed with 100% oxygen in an induction chamber and then euthanized by cervical dislocation. Quadriceps muscles were collected for metabolomics analyses.

### Global metabolomics

Non-targeted global metabolomic profiling of quadriceps muscles derived from 10% ribitol-treated, 10% Ribose-treated, and untreated P448L was performed by Metabolon (Durham, NC, USA), according to published methods (more detail in the Supplementary Information)^39^. In brief, neat methanol, containing select isotopically-labeled internal standards, was used to precipitate all the macromolecules (DNA, RNA, and protein) in the biological matrix. The purified supernatant was split into aliquots corresponding to the various analytical methodologies, then subsequently evaporated and reconstituted with the appropriate analytical injection solvent. Samples were analyzed with four separate methods: two separate reverse phase (RP)/UPLC-MS/MS methods with positive ion mode electrospray ionization (ESI), RP/UPLC-MS/MS method with negative ion mode ESI, and by HILIC/UPLC-MS/MS with negative ion mode ESI, The information output from the raw data files was automatically extracted and metabolites of known identity were recognized by comparison to metabolomic library entries of purified standards or recurrent unknown entities. This list of metabolites was further condensed to include only those that contained analytical values for each biological replicate, providing a total of 568 metabolites. Data are presented as fold change in three comparison groups: (1) Ribitol-treated versus untreated P448L control, (2) Ribose-treated versus untreatedP448L control, and (3) Ribose-treated versus Ribitol-treated P448L (metabolite ratio of > 1.00, significant (*p* ≤ 0.05, q ≤ 0.10) increase; metabolite ratio of < 1.00, significant (*p* ≤ 0.05, q ≤ 0.10) decrease).

### Statistical analysis and pathway diagrams

Statistical analysis of log-transformed metabolomic data was performed using Welch’s.

Two sample t test. For the post-hoc contrasts, p-values and false discovery rate (FDR) were calculated according to a previously proposed method^40^. Resulting q-values were assessed across the entire dataset and significance was defined as *p* ≤ 0.05 and q ≤ 0.10. Data are presented as box-and-whisker plots with Tukey whiskers that show mean (+), minimum, 25% quartile, median, 75% quartile, and maximum. Chemical structures were generated using the IUPAC International Chemical Identifier (InChI) in ACD/ChemSketch (Freeware) 2017.2.1.

## Electronic supplementary material

Below is the link to the electronic supplementary material.

Supplementary Material 1

## Data Availability

All data generated/analyzed in this study are included in this article or in the Supplementary Information files and can be provided upon request to M.P.C. (marcela.cataldi@atriumhealth.org) or Q.L.L (qi.lu@atriumhealth.org).
