# Supplementary material for: Ribitol and ribose treatments differentially affect metabolism of muscle tissue in FKRP mutant mice
Source: Sci Rep. 2025 Jan 8;15:1329. doi: 10.1038/s41598-024-83661-4 (PMC11711661; doi:10.1038/s41598-024-83661-4)
Supplement: Supplementary file 1 — Supplementary Material 1 [file 41598_2024_83661_MOESM1_ESM.pdf]

SUPPLEMENTARY INFORMATION

Ribitol and ribose treatments differentially affect metabolism of muscle tissue in FKRP mutant mice

Marcela P. Cataldi*, Qi L. Lu*

McColl-Lockwood Laboratory for Muscular Dystrophy Research, Atrium Health Musculoskeletal Institute, Carolinas Medical Center, 1000 Blythe Blvd. Charlotte, NC 28231, USA

Correspondence should be addressed to: Marcela P. Cataldi and Qi Long Lu, McColl-Lockwood Laboratory for Muscular Dystrophy Research, Carolinas Medical Center, Atrium Health, Charlotte, NC 28203, USA

Materials and Methods

Sample Accessioning: Following receipt, samples were inventoried and immediately stored at -80°C. Each sample received was accessioned into the Metabolon LIMS system and was assigned by the LIMS a unique identifier that was associated with the original source identifier only. This identifier was used to track all sample handling, tasks, results, etc. The samples (and all derived aliquots) were tracked by the LIMS system. All portions of any sample were automatically assigned their own unique identifiers by the LIMS when a new task was created; the relationship of these samples was also tracked. All samples were maintained at -80°C until processed.

Sample Preparation: Samples were prepared using the automated MicroLab STAR® system from Hamilton Company. Several recovery standards were added prior to the first step in the extraction process for QC purposes. To remove protein, dissociate small molecules bound to protein or trapped in the precipitated protein matrix, and to recover chemically diverse metabolites, proteins were precipitated with methanol under vigorous shaking for 2 min (Glen Mills GenoGrinder 2000) followed by centrifugation. The resulting extract was divided into five fractions: two for analysis by two separate reverse phase (RP)/UPLC-MS/MS methods with positive ion mode electrospray ionization (ESI), one for analysis by RP/UPLC-MS/MS with negative ion mode ESI, one for analysis by HILIC/UPLC-MS/MS with negative ion mode ESI, and one sample was reserved for backup. Samples were placed briefly on a TurboVap® (Zymark) to remove the organic solvent. The sample extracts were stored overnight under nitrogen before preparation for analysis.

Quality Assurance/Quality Control (QA/QC): Several types of controls were analyzed in concert with the experimental samples: a pooled matrix sample generated by taking a small volume of each experimental sample (or alternatively, use of a pool of well-characterized human plasma) served as a technical replicate throughout the data set; extracted water samples served

as process blanks; and a cocktail of QC standards that were carefully chosen not to interfere with the measurement of endogenous compounds were spiked into every analyzed sample, allowed instrument performance monitoring and aided chromatographic alignment. Tables 1 and 2 describe these QC samples and standards. Instrument variability was determined by calculating the median relative standard deviation (RSD) for the standards that were added to each sample prior to injection into the mass spectrometers. Overall process variability was determined by calculating the median RSD for all endogenous metabolites (i.e., non-instrument standards) present in 100% of the pooled matrix samples. Experimental samples were randomized across the platform run with QC samples spaced evenly among the injections.

Ultrahigh Performance Liquid Chromatography-Tandem Mass Spectroscopy (UPLC-MS/MS): All methods utilized a Waters ACQUITY ultra-performance liquid chromatography (UPLC) and a Thermo Scientific Q-Exactive high resolution/accurate mass spectrometer interfaced with a heated electrospray ionization (HESI-II) source and Orbitrap mass analyzer operated at 35,000 mass resolution. The sample extract was dried then reconstituted in solvents compatible to each of the four methods. Each reconstitution solvent contained a series of standards at fixed concentrations to ensure injection and chromatographic consistency. One aliquot was analyzed using acidic positive ion conditions, chromatographically optimized for more hydrophilic compounds. In this method, the extract was gradient eluted from a C18 column (Waters UPLC BEH C18-2.1x100 mm, 1.7 µm) using water and methanol, containing 0.05% perfluoropentanoic acid (PFPA) and 0.1% formic acid (FA). Another aliquot was also analyzed using acidic positive ion conditions, however it was chromatographically optimized for more hydrophobic compounds. In this method, the extract was gradient eluted from the same afore mentioned C18 column using methanol, acetonitrile, water, 0.05% PFPA and 0.01% FA and was operated at an overall higher organic content. Another aliquot was analyzed using basic negative ion optimized conditions using a separate dedicated C18 column. The basic extracts were gradient eluted from the column using methanol and water, however with 6.5mM Ammonium Bicarbonate at pH 8. The fourth aliquot was analyzed via negative ionization following elution from a HILIC column (Waters UPLC BEH Amide 2.1x150 mm, 1.7 µm) using a gradient consisting of water and acetonitrile with 10mM Ammonium Formate, pH 10.8. The MS analysis alternated between MS and data-dependent MS^n scans using dynamic exclusion. The scan range varied slighted between methods but covered 70-1000 m/z. Raw data files are archived and extracted as described below.

Bioinformatics: The informatics system consisted of four major components, the Laboratory Information Management System (LIMS), the data extraction and peak-identification software, data processing tools for QC and compound identification, and a collection of information interpretation and visualization tools for use by data analysts. The hardware and software foundations for these informatics components were the LAN backbone, and a database server running Oracle 10.2.0.1 Enterprise Edition.

Metabolon laboratory information management system (LIMS): The purpose of the Metabolon LIMS system was to enable fully auditable laboratory automation through a secure, easy to use, and highly specialized system. The scope of the Metabolon LIMS system encompasses sample accessioning, sample preparation and instrumental analysis and reporting and advanced data analysis. All of the subsequent software systems are grounded in the LIMS data structures. It has been modified to leverage and interface with the in-house information extraction and data visualization systems, as well as third party instrumentation and data analysis software.

Data Extraction and Compound Identification: Raw data was extracted, peak-identified and QC processed using Metabolon's hardware and software. These systems are built on a web-

service platform utilizing Microsoft’s .NET technologies, which run on high-performance application servers and fiber-channel storage arrays in clusters to provide active failover and load-balancing. Compounds were identified by comparison to library entries of purified standards or recurrent unknown entities. Metabolon maintains a library based on authenticated standards that contains the retention time/index (RI), mass to charge ratio (*m/z)*, and chromatographic data (including MS/MS spectral data) on all molecules present in the library. Furthermore, biochemical identifications are based on three criteria: retention index within a narrow RI window of the proposed identification, accurate mass match to the library +/- 10 ppm, and the MS/MS forward and reverse scores between the experimental data and authentic standards. The MS/MS scores are based on a comparison of the ions present in the experimental spectrum to the ions present in the library spectrum. While there may be similarities between these molecules based on one of these factors, the use of all three data points can be utilized to distinguish and differentiate biochemicals. More than 3300 commercially available purified standard compounds have been acquired and registered into LIMS for analysis on all platforms for determination of their analytical characteristics. Additional mass spectral entries have been created for structurally unnamed biochemicals, which have been identified by virtue of their recurrent nature (both chromatographic and mass spectral). These compounds have the potential to be identified by future acquisition of a matching purified standard or by classical structural analysis.

Curation: A variety of curation procedures were carried out to ensure that a high quality data set was made available for statistical analysis and data interpretation. The QC and curation processes were designed to ensure accurate and consistent identification of true chemical entities, and to remove those representing system artifacts, mis-assignments, and background noise. Metabolon data analysts use proprietary visualization and interpretation software to confirm the consistency of peak identification among the various samples. Library matches for each compound were checked for each sample and corrected if necessary.

Metabolite Quantification and Data Normalization: Peaks were quantified using area-under-the-curve. For studies spanning multiple days, a data normalization step was performed to correct variation resulting from instrument inter-day tuning differences. Essentially, each compound was corrected in run-day blocks by registering the medians to equal one (1.00) and normalizing each data point proportionately (termed the “block correction”; Figure 2). For studies that did not require more than one day of analysis, no normalization is necessary, other than for purposes of data visualization. In certain instances, biochemical data may have been normalized to an additional factor (e.g., cell counts, total protein as determined by Bradford assay, osmolality, etc.) to account for differences in metabolite levels due to differences in the amount of material present in each sample.

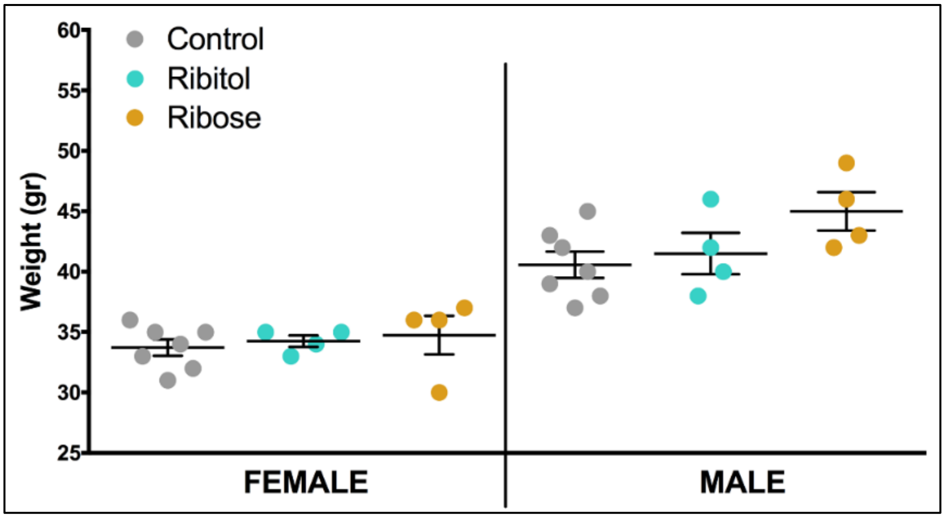

Figure S1. Body weight (gr, grams) comparison among mice treated with 10% ribitol or 10% ribose in drinking water, and age-matched control mice at 30 weeks of age. (n= 7 for control cohort; n=4 for treated cohorts).

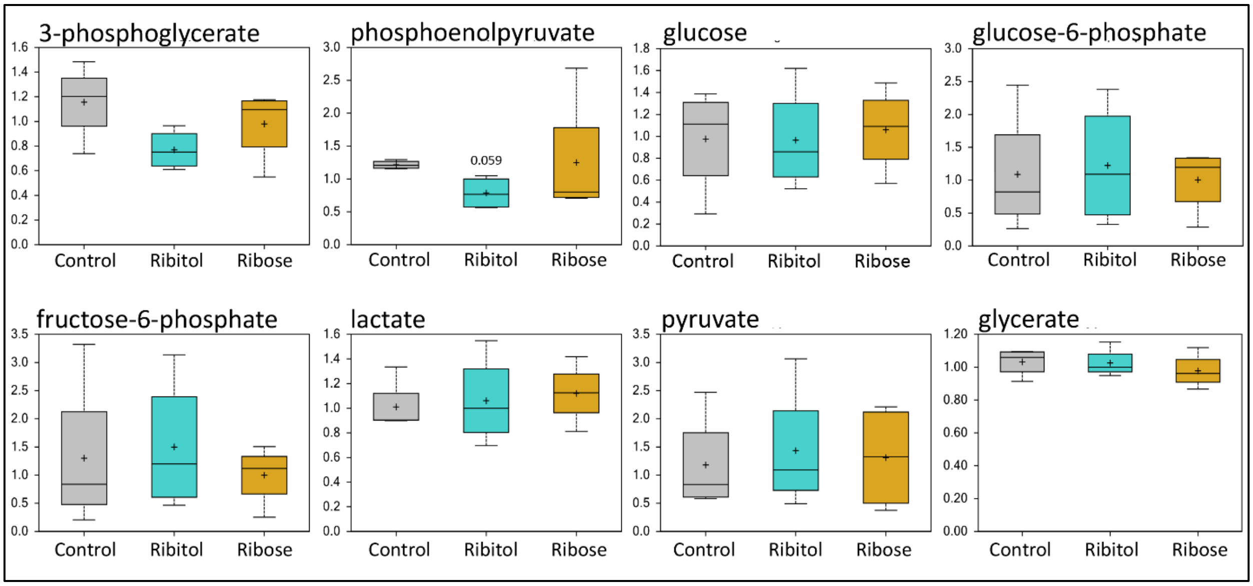

Figure S2. Carbohydrate Metabolism. Glycolysis pathway. Comparison of metabolite abundances in quadriceps from 32-week-old control P448L mice, and 10% ribitol or 10% ribose treated P448L mice. Vertical axis represents scaled intensity in arbitrary units. * p≤ 0.05, ** p≤ 0.01, *** p≤ 0.001 compared to the control, as determined by Welch's two-sample t-Test.

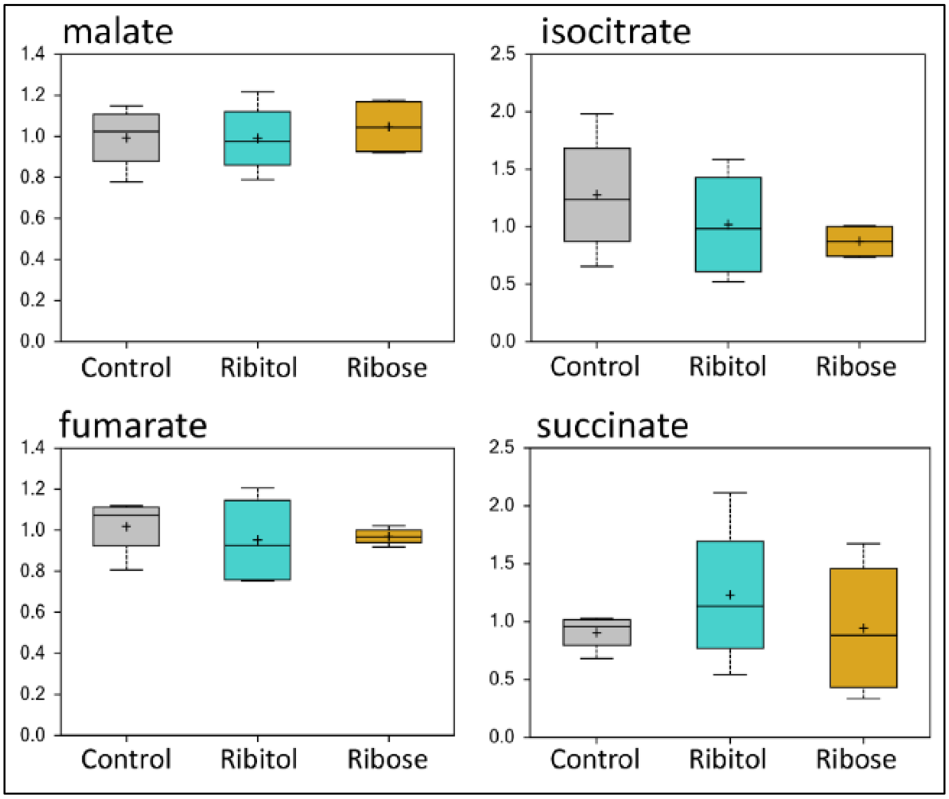

Figure S3. TCA Cycle. Comparison of metabolite abundances in quadriceps from 32-week-old control P448L mice, and 10% ribitol or 10% ribose treated P448L mice. Vertical axis represents scaled intensity in arbitrary units.

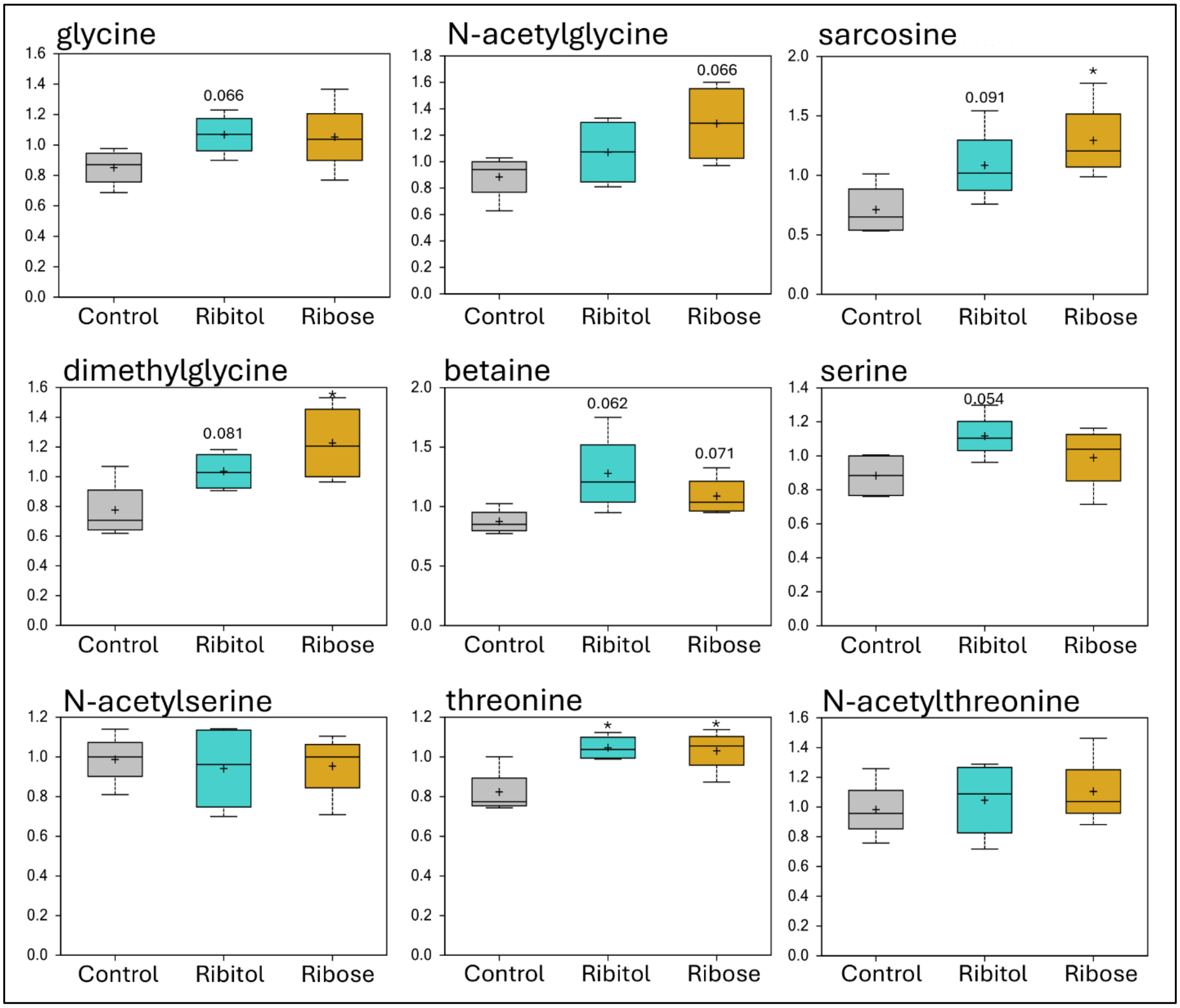

Figure S4. Amino Acid Pathway. Glycine, Serine and Threonine Metabolism. Comparison of metabolite abundances in quadriceps from 32-week-old control P448L mice, and 10% ribitol or 10% ribose treated P448L mice. Vertical axis represents scaled intensity in arbitrary units. * $p \leq 0.05$ compared to the control as determined by Welch's two-sample t-Test.

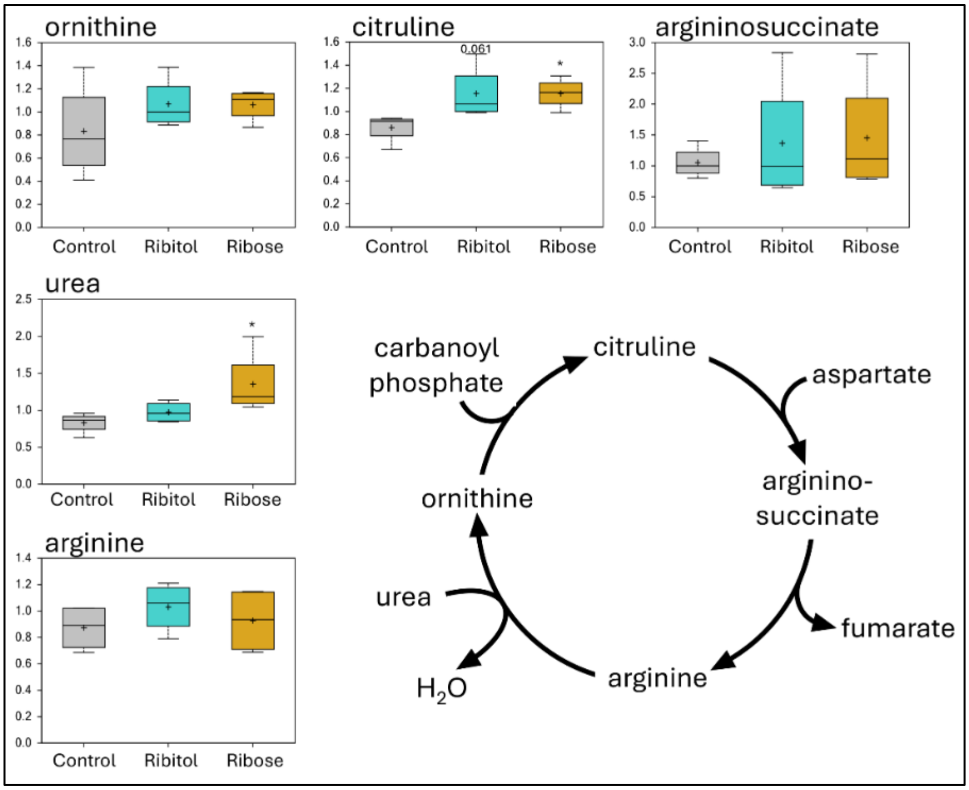

Figure S5. Urea cycle. Comparison of metabolite abundance in quadriceps from 32-week-old control P448L mice and 10% ribitol or 10% ribose treated P448L mice. Vertical axis represents scaled intensity in arbitrary units. * p≤ 0.05 compared to the control as determined by Welch's two-sample t-Test.

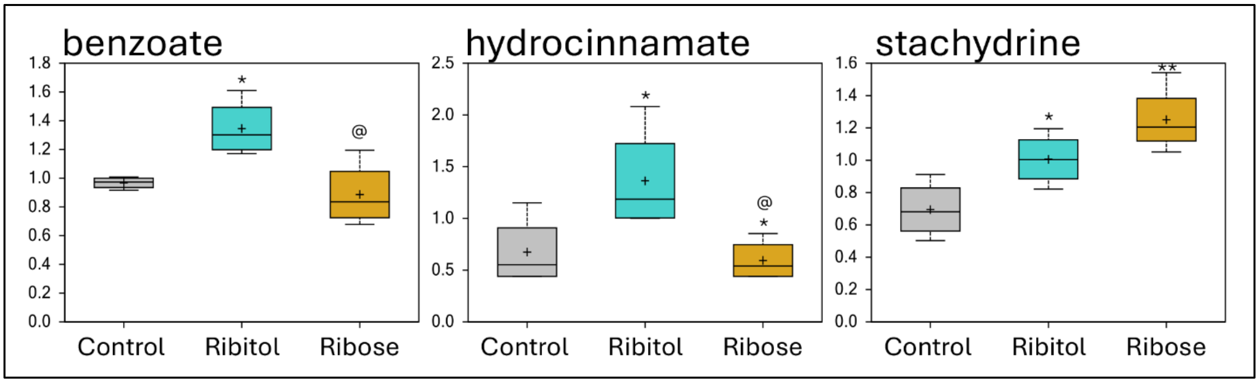

Figure S6. Xenobiotics metabolism. Comparison of metabolite abundance in quadriceps from 32-week-old control P448L mice and 10% ribitol or 10% ribose treated P448L mice. Vertical axis represents scaled intensity in arbitrary units. * $p \leq 0.05$, ** $p \leq 0.01$, *** $p \leq 0.001$ compared to the control. @ $p \leq 0.05$, @@ $p \leq 0.01$, @@@ $p \leq 0.001$ compared to ribitol treatment, as determined by Welch's two-sample t-Test.

Metabolic Pathway	Sub Pathway	Metabolite	[a]HMDB	Fold of Change		
				Ribitol vs Control	Ribose vs Control	Ribose vs Ribitol
Amino Acid	Glycine, Serine and Threonine Metabolism	sarcosine	HMDB00271	1.52*	1.82	1.19
		dimethylglycine	HMDB00092	1.34*	1.58	1.18
		threonine	HMDB00167	1.27	1.25	0.98
	Alanine and Aspartate Metabolism	alanine	HMDB00161	1.03	1.14	1.11
	Glutamate Metabolism	glutamate	HMDB00148	1.24*	1.33	1.08
	Histidine Metabolism	1-methylhistidine	HMDB00001	0.44*	0.58	1.33
	Lysine Metabolism	5-aminovalerate	HMDB03355	2.59	2.19*	0.84
	Methionine, Cysteine, SAM and Taurine Metabolism	methionine	HMDB00696	1.21	1.25	1.03
		S-methylmethionine	HMDB38670	2.59	3.32*	1.28
		methionine sulfoxide	HMDB02005	0.99	1.22	1.23
		hypotaurine	HMDB00965	3.32	1.74	0.52
	Urea cycle; Arginine and Proline Metabolism	urea	HMDB00294	1.17	1.63	1.39
		citrulline	HMDB00904	1.34*	1.34	1.00
	Guanidino and Acetamido Metabolism	1-methylguanidine	HMDB01522	0.62*	1.52*	2.45
	Glutathione Metabolism	4-hydroxy-nonenal-glutathione	n/a	1.26	1.09	0.86
Carbohydrate	Pentose Phosphate Pathway	ribitol-5-phosphate	n/a	3.27*	2.94	0.90
	Pentose Metabolism	ribitol	HMDB00508	40.42	44.13	1.09
		ribonate	HMDB00867	6.09	117.79	19.36
		arabonate/xylonate	n/a	1.03	0.64	0.62*
	Fructose, Mannose and Galactose Metabolism	galactonate	HMDB00565	0.71*	0.52	0.74
	Nucleotide Sugar	CDP-ribitol	n/a	13.19	17.11	1.30
	Aminosugar Metabolism	N-glycolylneuraminate	HMDB00833	0.64	0.62	0.97
	Advanced Glycation End-product	N6-carboxymethyllysine	n/a	1.03	1.78*	1.72
Energy	TCA Cycle	citrate	HMDB00094	1.37	1.49*	1.08
		succinylcarnitine (C4-DC)	HMDB61717	0.78	1.18	1.51
Lipid	Fatty Acid Metabolism (also BCAA Metabolism)	propionylcarnitine (C3)	HMDB00824	1.41	1.34	0.95
	Fatty Acid Metabolism (Acyl Carnitine, Medium Chain)	hexanoylcarnitine (C6)	HMDB00705	0.89	0.47	0.52
		octanoylcarnitine (C8)	HMDB00791	0.96	0.44	0.46
		decanoylcarnitine (C10)	HMDB00651	0.87	0.50	0.58
	Fatty Acid Metabolism (Acyl Carnitine, Monounsaturated)	5-dodecenoylcarnitine (C12:1)	HMDB13326	1.03	0.56	0.54
	Phosphatidylcholine (PC)	1-oleoyl-2-linoleoyl-GPC (18:1/18:2)	n/a	1.24	1.03	0.83
	Lysophospholipid	1-palmitoleoyl-GPC (16:1)	HMDB10383	1.38	1.14	0.83
		1-oleoyl-GPC (18:1)	HMDB02815	1.50	1.27	0.85
		1-linoleoyl-GPC (18:2)	HMDB10386	1.45	1.04	0.71
		1-oleoyl-GPE (18:1)	HMDB11506	1.56	1.25	0.81
		1-linoleoyl-GPE (18:2)	HMDB11507	1.48*	1.12	0.75
	Glycerolipid Metabolism	glycerol 3-phosphate	HMDB00126	0.71	0.40	0.57
	Diacylglycerol	palmitoyl-linoleoyl-glycerol (16:0/18:2)	n/a	0.84	1.12	1.33
		linoleoyl-linolenoyl-glycerol (18:2/18:3)	n/a	0.75	1.05	1.41
		stearoyl-docosahexaenoyl-glycerol (18:0/22:6)	n/a	0.71	0.89	1.25
	Sphingolipid Synthesis	sphingadienine	n/a	2.37	2.21	0.93
	Ceramides	N-stearoyl-sphingosine (d18:1/18:0)	HMDB04950	0.88	0.69	0.78
		ceramide (d18:1/17:0, d17:1/18:0)	n/a	0.89	0.61	0.68
Nucleotide	Purine Metabolism, (Hypo)Xanthine/Inosine containing	AICA ribonucleotide	HMDB01517	2.03	12.92	6.36
		inosine	HMDB00195	1.67	1.61	0.97
		xanthine	HMDB00292	1.40	1.60	1.14
	Pyrimidine Metabolism, Orotate containing	orotidine	HMDB00788	2.36	3.62	1.53*
	Pyrimidine Metabolism, Uracil containing	2'-deoxyuridine	HMDB00012	0.68	0.72	1.07
Cofactors and Vitamins	Nicotinate and Nicotinamide Metabolism	nicotinamide	HMDB01406	1.06	1.26	1.19*
		nicotinamide riboside	HMDB00855	1.70	2.45	1.44
		nicotinamide N-oxide	HMDB02730	2.90*	4.91	1.69
		N1-Methyl-2-pyridone-5-carboxamide	HMDB04193	1.67	3.35	2.01
		N1-Methyl-4-pyridone-3-carboxamide	HMDB04194	1.71	3.01	1.76*
	Thiamine Metabolism	thiamin (Vitamin B1)	HMDB00235	1.24	2.48	2.00
		thiamin diphosphate	HMDB01372	0.59	0.60	1.01
	Vitamin B6 Metabolism	pyridoxamine phosphate	HMDB01555	1.29	1.46	1.13
Xenobiotics	Benzoate Metabolism	benzoate	HMDB01870	1.39	0.92	0.66
		3-phenylpropionate (hydrocinnamate)	HMDB00764	2.02	0.88	0.44
	Food Component/Plant	stachydrine	HMDB04827	1.45	1.80	1.24

Table S1. Summary of all the metabolites that achieve statistically significant difference ($p \leq 0.05$) between the groups shown. Red and green indicate upregulated or downregulated metabolites, respectively. [a] Human metabolome database (www.hmdb.ca). “*” indicates p value of $0.05 < p < 0.10$.

Metabolic Pathway	Sub Pathway	Metabolite	[a]HMDB	Fold of Change		
				Ribitol vs Control	Ribose vs Control	Ribose vs Ribitol
Peptide	Gamma-glutamyl Amino Acid	gamma-glutamylleucine	HMDB11171	1.05	1.26*	1.20
		gamma-glutamyl-alpha-lysine	n/a	1.28	1.23*	0.96
		gamma-glutamyl-epsilon-lysine	HMDB03869	0.59	1.60	2.71
		gamma-glutamylvaline	HMDB11172	0.99	1.10	1.11
	Dipeptide	alanylleucine	HMDB28691	1.43	1.39	0.97
		glutaminylleucine	n/a	1.23	1.56	1.27
		glycylleucine	HMDB00759	1.20	1.39	1.16
		glycylvaline	HMDB28854	1.14	1.22	1.07
		leucylalanine	HMDB28922	1.06	1.14	1.07
		leucylglycine	HMDB28929	1.29	1.34	1.03
		phenylalanylglycine	HMDB28995	1.17	1.00	0.86
		prolylglycine	HMDB11178	1.16	1.41	1.22
		valylglutamine	HMDB29125	1.72*	2.58*	1.50
		valylglycine	HMDB29127	1.33	1.72*	1.30
		valylleucine	HMDB29131	1.46	1.33	0.91
		leucylglutamine*	HMDB28927	1.48*	1.44	0.97
	Acetylated Peptides	phenylacetylglycine	HMDB00821	1.32	16.96	12.82

Table S2. Peptide pathway. Comparison of metabolite abundance between 10% ribitol or 10% ribose treated mice and control, as well as between two treated groups. [a] Human metabolome database (). "*" indicate upregulated metabolites approaching significance ($0.05<p<0.10$) as determined by Welch's two-sample t-test.
